# A participatory epidemiological study of major cattle diseases amongst Maasai pastoralists living in wildlife-livestock interfaces in Maasai Mara, Kenya

**DOI:** 10.1007/s11250-018-01790-1

**Published:** 2019-01-25

**Authors:** Daniel Nthiwa, Silvia Alonso, David Odongo, Eucharia Kenya, Bernard Bett

**Affiliations:** 1 Department of Biological Sciences, University of Embu, P. O BOX 6, Embu, 60100 Kenya; 2 International Livestock Research Institute, (ILRI), P. O BOX 30709, Nairobi, 00100 Kenya; 3 International Livestock Research Institute, (ILRI), P. O BOX 5689, Addis Ababa, Ethiopia; 4 School of Biological Sciences, University of Nairobi, P. O BOX 30197, Nairobi, 00100 Kenya

**Keywords:** Participatory techniques, Cattle diseases, Maasai pastoralists, Livelihoods

## Abstract

Livestock-wildlife interactions promote the transmission of a wide range of infectious diseases that constraint livestock production. We used a participatory appraisal approach to find out and rank infectious diseases of concern to pastoralists in a zone of intense wildlife-livestock interaction and another zone with limited interactions. Four villages were selected purposively in areas with intensive cattle-wildlife interactions (zone 1), and another two in areas with low to moderate cattle-wildlife interactions (zone 2). Data were collected in focus group discussions (FGDs) using participatory epidemiological methods (PE); each group had 8–13 participants. Results of impact matrix scoring from all sites indicated that malignant catarrhal fever (MCF), anthrax, foot and mouth disease (FMD), contagious bovine pleuropneumonia (CBPP), east coast fever (ECF) and African animal trypanosomiasis (ATT), in decreasing order, had the highest impact on livestock production. A Kruskal–Wallis test revealed a significant difference in FMD annual prevalence between cattle age groups (*p* < 0.001) and was the highest in animals > 4 years (median score of 32.5, range, 10–50). FMD had the highest impact on milk production, but based on veterinary costs (treatment costs), it was ranked second to CBPP. The study provides information on disease priorities that occur in the target zones in Mara ecosystem and which the local pastoralists must consider when accessing key ecosystem services such as water and pasture.

**Electronic supplementary material:**

The online version of this article (10.1007/s11250-018-01790-1) contains supplementary material, which is available to authorized users.

## Introduction

Livestock production is the main source of livelihoods and nutrition for over 300 million people residing in sub-Saharan Africa (Abdilatif et al. 2018). In Kenya, it also contributes significantly to income, food and livelihood resilience of pastoral communities (Smith et al. 2013). For many years, the Maasai pastoralists in Kenya have raised their livestock in wildlife inhabited areas where livestock-wildlife interactions occur mainly at watering and grazing areas (Bedelian and Ogutu 2017). This has been the predominant production system for many years, but of late, there has been a shift towards the establishment of wildlife conservancies to support better utilization of the rangelands, both for livestock farming and wildlife conservation (Løvschal et al. 2017). The revenues generated from wildlife-related tourism contribute a large proportion of the household income in these communities (Bedelian and Ogutu 2017).

While the establishment of wildlife conservancies has been recognized as a sustainable intervention for protecting wildlife and their ecosystems in areas with intense livestock-wildlife interactions, several challenges including the competition for resources and increased livestock-wildlife interactions, leading to increased transmission of infectious diseases, have been identified. Examples of animal health threats associated with wildlife in these locations include foot and mouth disease (FMD) and zoonosis such as brucellosis and leptospirosis. Several studies have also demonstrated a substantial decline in wildlife populations due to increased competition for resources and degradation of ecosystems (Ogutu et al. 2016). Such decline of wildlife populations might also increase infectious disease transmission through indirect disease transmission processes. This is because a decline in animals that act as dead-end hosts for pathogens or those that limit contact between susceptible and infectious hosts erode the “dilution effect” that is thought to limit disease emergence in stable ecosystems (Huang et al. 2016).

This study aimed to determine the impact of livestock diseases on the livelihoods of the Maasai pastoralists in zones with varied degree of livestock-wildlife interactions. It specifically investigated whether the distribution of livestock diseases varied with the degree of livestock-wildlife interactions. These findings will help understand threats to animal health and livelihoods in the area and support prioritization of interventions to enhance cattle productivity and improve livelihoods and nutritional security for the Maasai pastoralists.

## Materials and methods

### Study area

**Fig. 1:**
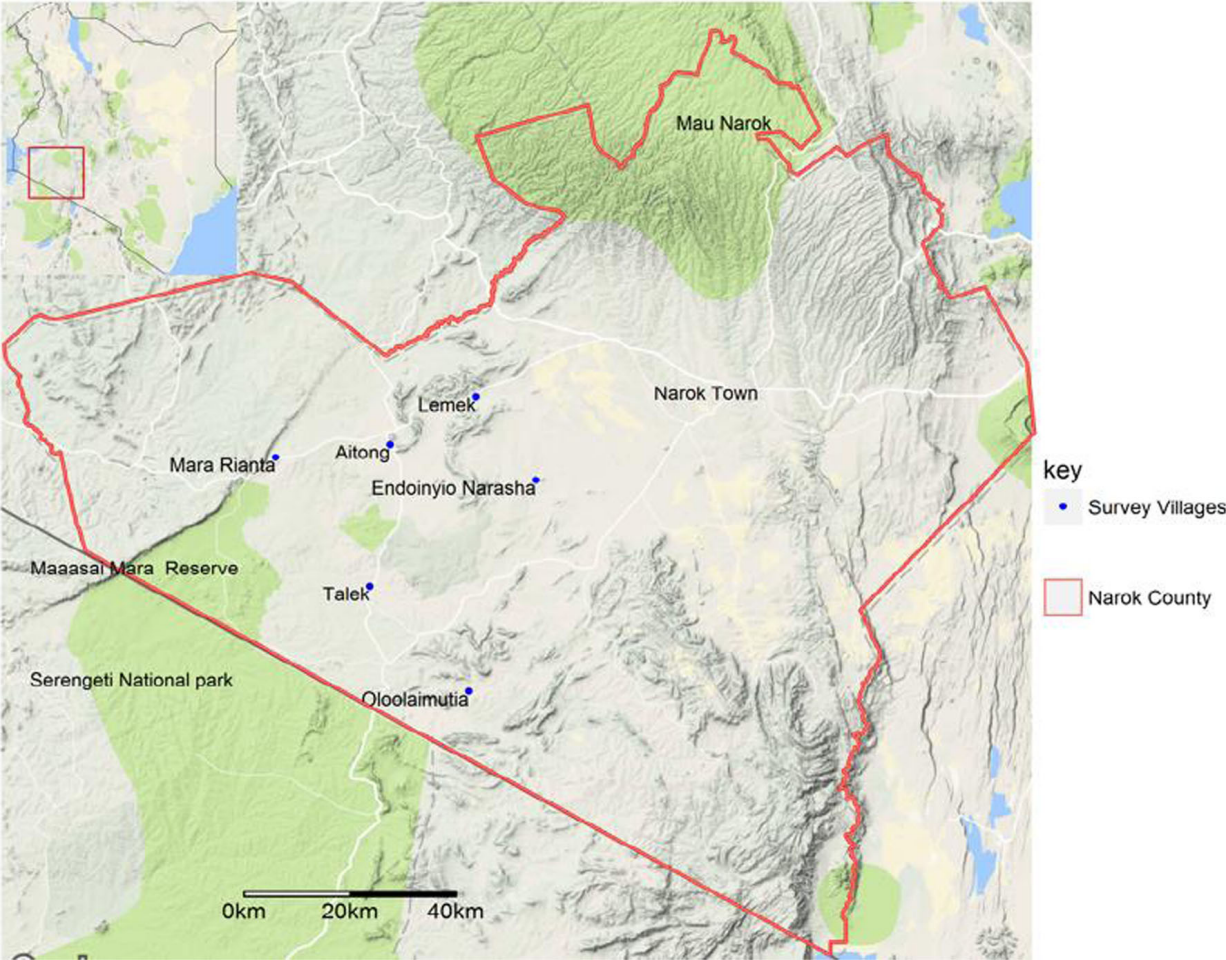
Map of Maasai Mara ecosystem showing the location of survey sites

This study was conducted in Maasai Mara ecosystem in South Western Kenya (Fig.1). The area consisted of the Maasai Mara National Reserve (MMNR) and the adjacent territories co-inhabited by pastoralists, livestock and wildlife (Bedelian and Ogutu 2017).

The study area was stratified by land use type into two zones; the first (zone 1) bordered the MMNR and wildlife conservancies and therefore had intensive livestock-wildlife interactions, while the second (zone 2) was more than 40 km away from the MMNR and had low to moderate livestock-wildlife interactions, more intensive cattle production and some crop cultivation. The defined zones provided an ecological gradient that allowed analyses to be made on the risk of livestock diseases associated with land use change. People that lived in zone 1 utilized conservancies and MMNR for dry season grazing while those from zone 2 had largely adopted sedentary grazing lifestyles.

### Study design and selection of villages

The study used a cross-sectional study design that involved four villages in zone 1 (i.e. Talek, Mara Rianta, Oloolaimutia and Aitong [approximately 10 km away from the MMNR]) and two villages in zone 2 (i.e. Lemek and Endoinyio-Narasha villages). These villages were selected purposefully to provide the required ecological gradient for the study.

### Data collection

The study was conducted between September and October 2016. Focus group discussions (FGDs) were done in each target village. Preliminary visits to selected villages were planned with local authorities to schedule meetings with the farmers and introduce the project and its objectives. Purposive selection of participants was done to identify people who could provide reliable information on diseases and other ecosystem services in the area. Participants had to be 18 years old and above, residents in the village and either own livestock or come from households that kept livestock. FGDs were separated by gender in all the villages. In total, the number of FGDs conducted in zones 1 and 2 were 8 and 4 respectively, while based on gender, there were 6 FGDs for both men and women groups. Each discussion group comprised 8–13 participants and discussions were conducted in the local Maasai language with the assistance of a translator. The discussions were guided by a checklist of open-ended questions that were pre-tested in three villages (not included in the main study) within Mara ecosystem. The questionnaire checklist was refined before the start of the work. The participatory epidemiological tools used to collect data included semi-structured interviews, pair-wise ranking, disease impact matrix scoring and proportional piling (Catley et al. 2012). During the FGDs, probing was used to ensure consistency of information obtained and to provide detailed information on items being discussed. Voice recorders were used to capture all discussions and to supplement notes taken at the time. Each group discussion lasted for at least 1.5 h. Key questions addressed included livestock species kept and their respective benefits; livestock diseases and their impacts on livelihoods from livestock, and foot and mouth disease (FMD) as an example of MMNR ecosystem disservice.

### Herd species composition

Using proportional piling technique, FGD groups were asked to list the livestock species kept in the community and indicate the relative sizes of each species in an average herd. To do this, livestock species were listed on a flip chart and the group given 100 stones to distribute across species based on their relative abundance (i.e. the most abundant species received the highest number of stones). Stones allocated to each species were counted and participants were notified of the scores. Probing was used to discuss the numbers and to understand the reasons that informed the allocations made and for herd composition.

### Livestock benefits and disease constraints to cattle production

Participants were asked to identify benefits they derived from each listed livestock species. Proportional piling was used with 100 stones as described above, to determine the relative importance of each benefit to households’ livelihoods. The number of stones allocated to each benefit was determined and discussions held to determine the reasons for the results of the exercise.

Participants were also asked to list the most common cattle diseases they had observed in their herds in the previous year (i.e. between September 2015 and October 2016). Using the list generated, pair-wise ranking was used (comparing two diseases at a time) to identify the one that was more common in livestock in the reference period. To help complete this exercise, a simple matrix that had disease names on the *X* and *Y* axes was designed. A disease that was perceived to be prevalent received the highest scores. Probing was used to identify reasons that underlined the observations made.

### FMD annual age-specific prevalence and mortality

FMD was used as a case study disease to determine perceptions on how cattle-wildlife interactions affect animal disease prevalence. Proportional piling was used to estimate annual age-specific prevalence and mortalities due to FMD. Groups were asked to categorize cattle in a herd into various age groups using Maasai local names. The identified age groups were written on a flip chart and the group provided with 100 stones (representing herd size) to allocate them to age group based on their relative sizes. Using the scores allocated to each age group, participants were asked to further divide the stones into two piles representing the proportion of animals that remained healthy in the past year versus those that got FMD. Each pile of stones corresponding to FMD-infected cattle was further subdivided to show the proportion that survived the infection and those that died (case fatality). This exercise was repeated for all age groups and probing done to determine reasons that supported the scores allocated to each pile.

The market value of cattle with and without FMD was estimated. Each group was asked to give the prices of cattle when healthy and when infected for each age group. The prices obtained were based on group consensus.

### Impacts of livestock diseases on livelihoods from livestock

The impact that each of the identified disease had on the livestock-associated benefits was determined using disease impact matrix scoring. A matrix comprising prioritized diseases on the *X*-axis and livestock benefits on the *Y*-axis was developed. The exercise started with the ranking of the benefits by importance along the *Y*-axis; a benefit that was highly ranked received more scores than those that were not. Thereafter, we distributed the scores already apportioned to each benefit across the diseases along the *X*-axis; a disease that had the highest impact on a given benefit received higher scores than those that did not. The overall disease impact scores were derived by adding up all the scores that each disease got. This approach allowed the generation of weighted scores as diseases that had the highest impact on a benefit that was highly preferred was identified as being important in the area.

Finally, the relative costs of treating FMD compared to the other listed diseases were indicated using 100 stones. Scores of 0 and 100 represented very low and very high veterinary costs, respectively.

### Data management and analysis

Semi-quantitative data obtained from scoring and ranking exercises were entered into a database designed using MS Excel (Microsoft® Excel, Washington, 2013) and exported into Statistical Package for Social Sciences (SPSS), version 22.0 (Corp 2013) for analysis using non-parametric and descriptive statistical methods. Analysis involved computing percentages, medians and ranges of the scores. Kendall’s coefficient of concordance (*W*) was used to assess the level of agreement between groups as follows: weak agreement, *W* < 0.26, *p* > 0.05; moderate agreement, *W* = 0.26–0.38, *p* < 0.05; strong agreement, *W* > 0.38, *p* < 0.01 (Ayele et al. 2016). Kruskal–Wallis test was used to compare the median scores between zones, gender and diseases.

## Results

### Livestock species

Sheep, cattle, goats, chicken and donkeys were identified by all the groups as the common livestock species kept in the target zones (Table 1). Their relative proportions determined from proportional piling exercises did not differ by gender of participants or zone as indicated by the strong overall degree of agreement between groups (Kendall’s *W =* 0.99, *p* = < 0.001, *n* = 12 FGDs). The proportions of sheep and cattle were perceived to be higher in both zones compared to those of the other livestock species. Reasons given for the higher preference for sheep included better drought tolerance, steady production of milk even when cattle had been moved to dry season grazing areas in search of pastures, could be slaughtered at home, reproduced frequently—at least twice annually, and could be given out as gifts. Cattle, on the other hand, provided more income compared to sheep and were sold for capital expenses.

**Table 1:** Median scores and their respective ranges obtained from ranking livestock species kept by the Maasai in Mara ecosystem, Kenya

	Livestock species				
Zones	Cattle	Sheep	Goats	Chicken	Donkey
Zone 2^a^	27.5 (25, 33)	38 (35, 44)	20 (15, 20)	9 (7, 12)	5 (4, 6)
Zone 1^b^	27.5 (21, 32)	41 (34, 45)	18.5 (16, 20)	10 (8, 20)	5 (3, 8)
Overall scores (*n* = 12)	27.5 (21, 33)	39.5 (34, 45)	19 (15, 20)	9.5 (7, 20)	5 (3, 8)

*n*, number of FGDs (12) that participated in the proportional piling

^a^Area with low to moderate cattle-wildlife interactions

^b^Area with intense cattle-wildlife interactions

The relative proportions of goats, donkeys and chicken were lower than those of sheep and cattle. Goats were perceived to be more susceptible to diseases such as “olodua” (used for both enterotoxemia or peste des petits ruminants [PPR]), “olomorooj” (goat pox) and “orkipei” (contagious caprine pleuropneumonia [CCPP]). Donkeys were used for draught power to transport water and firewood, and were more likely to be stolen than the other livestock species. Predation and diseases were identified as the main challenges in poultry production.

### Ranking of benefits from livestock

Table 2 gives overall median scores on perceived benefits from livestock by zone. In descending order of importance, the study identified (i) income from sale of livestock, (ii) milk, (iii) employment, (iv) payment of bride price, (v) meat and (vi) social status associated with livestock ownership as the most important livestock livelihoods. Other benefits such as hides for clothing and the use of livestock for draught power were regarded as being least important. A Kruskal–Wallis test comparison of the median scores for the livelihoods from livestock showed no statistically significant differences by zone (*p* > 0.05), apart from meat consumption (*p* = 0.006) which was apparently higher in zone 2 than zone 1. All the groups had high level of concordance on the generated median scores for the benefits (*W* = 0.78; range, 0.54–1.0; *n* = 12 FGDs) and there were no gender differences observed.

**Table 2:** Overall relative importance of livestock benefits, results obtained by proportional piling

Village		Benefits								
		Milk consumption	Meat consumption	Income	Bride price	Social status	Investment	Employment	Draught power	Hides
Zone 2^a^	Md (Mn-Mx)	23 (17-30)	11 (10-16)	33 (32-50)	10.5 (7-20)	6 (4-14)	_d_	21 (0-21)	3 (1-6)	3 (3-3)
	*N*	4	4	4	4	3		1	4	2
Zone 1^b^	^c^Md (Mn-Mx)	20 (6-32)	6.5 (2-9)	34 (23-68)	14 (5-18)	7 (1-12)	17 (0-17)	12 (4-21)	4 (1-9)	3 (2-3)
	*N*	8	8	8	7	8	1	6	6	4
Total	Md (Mn-Mx)	21 (6-32)	8 (2-16)	33 (23-68)	11 (5-20)	7 (1-14)	17 (0-17)	12 (4-21)	3 (1-9)	3 (2-3)
	*N*	12	12	12	11	11	1	7	10	6

*n*, number of FGDs that contributed data to that benefit. Median scores for investment and employment as a benefit was not compared between villages, gender and zones due to few cases

^a^Area with low to moderate cattle-wildlife interactions

^b^Area with intense cattle-wildlife interactions

^c^*Md*, median; *Mn*, minimum; *Mx*, maximum

^d^Dash (-) means that the benefit was not mentioned hence not included in proportional piling

### Prioritization of livestock diseases

The participants identified MCF, ECF, FMD, CBPP and AAT as five most prevalent diseases that affected cattle in the area in the previous year. Bovine ephemeral fever (BEF), anthrax, pox, salmonellosis (“orsetet”) and diseases with nervous syndrome such as bovine cerebral theileriosis (BCT), locally called “ormilo”, were least prevalent. No differences were noted on the spectra of diseases reported between zones and by gender.

### FMD prevalence, mortality and impacts on market value

The groups identified three main cattle age groups, namely, calves (“elasho” < 1 year), weaners (“olaram” 2–3 years) and mature adults (“nkishu sapukin” > 4 years). The overall median proportion of cattle in the various age groups over the last 1 year showed that, those above 4 years constituted the largest percentage in the herd structure with 50% (range, 45–70). The calves and weaners were 20% (10–30) and 30% (20–30), respectively. The annual median prevalence of FMD was the highest amongst cattle > 4 years with 32.5% (range, 10–50), against 18.5% (10–25) and 12.5% (7–25) in weaners and calves respectively. A Kruskal–Wallis test comparison of these median scores indicated a significant difference in FMD annual prevalence between the cattle age groups (*p* < 0.001), but not between weaners and calves.

Slightly higher mortalities associated with FMD were observed amongst calves with median scores of 4.5% (range, 2–15) compared to weaners (0.5%; 0–10) and mature adults (1%; 0–15). The annual age-specific median prevalence and mortality estimates for FMD did not differ significantly between gender. All the 12 FGDs had strong level of agreement (*W* = 0.76, *p* < 0.001) for estimates on herd structure and annual age-specific FMD prevalence. The median scores for FMD prevalence and mortality estimates between zones were only significant for mortality estimates (Kruskal–Wallis test, *p* = 0.041). The median scores for FMD-associated mortalities in cattle were, respectively, 3.0% (range, 0–15) and 0.0% (range; 0–5) in zones 1 and 2, while the annual prevalence estimates were 18% (range, 7–50) and 20% (range, 10–50) respectively.

The median value of healthy cattle (without FMD) was US$ 300 (range, 250–700) for mature adults compared to weaners (US$ 200, 100–300) and calves (US$ 100, 50–175). The reduction in market sale value of FMD-infected cattle was estimated to be the highest in mature adults (median losses of US$ 175, range, 75–370) while value of calves and weaners would decrease by US$ 45 (range, 20–100) and US$ 60 (range, 20–150) of its normal value respectively. There were no significant differences between zones and gender on the given prices of cattle when healthy and when FMD infected. The overall agreement between groups for the given prices was high (*W* = 0.92, *p* < 0.001, *n* = 12 FGDs).

### Disease impact matrix scoring results

Disease impact matrix scoring technique was used to rank cattle diseases based on their impacts on the livestock benefits. Reported impacts included reduction on milk production, decline in income from sale of animals and increased veterinary costs (supplementary material 1). All the diseases identified were perceived to reduce milk production. These diseases were ranked in a descending order based on their impacts on milk production as FMD, CBPP, AAT and MCF. This ranking was consistent between groups (*W* = 0.49, *p* < 0.001, *n* = 12); no significant differences were therefore observed on these impact scores by zone and gender. The impact of AAT was associated with frequent infections, while MCF was associated with high mortalities in livestock including those that were lactating.

MCF, CBPP, FMD, anthrax and goat pox, in decreasing order, were perceived to reduce income from the sale of live animals. Participants indicated that MCF, pox and anthrax were difficult to control, and therefore caused extensive case fatalities, because of unavailability of drugs and vaccines. Both FMD and CBPP reduced income and milk as they infected many cattle within herds with high case fatality.

## Discussion

This study used participatory epidemiological (PE) tools to prioritize diseases that affect cattle herds in Mara ecosystem. PE tools have been widely used by researchers to investigate animal health related topics in resource poor areas (Kimaro et al. 2017; Abdilatif et al. 2018). In this study, our results showed that livestock production contributes significantly to the livelihoods of the Maasai, consistent with other findings (Bellet et al. 2012; Ayele et al. 2016). The main livestock-derived benefits identified by participants are aligned with findings reported in previous studies (Jibat et al. 2013). Sheep and cattle were prioritized as the most important domestic species that sustained households’ livelihood in the area. These species were kept in large stocks as a strategic tool to sustain milk and meat production, which constitute an important diet for pastoralists (Smith et al. 2013). However, with increasingly recurrent droughts in the area, sheep were currently a more preferred livestock species as they performed better than cattle in drought situations and where feeds are scarce.

MCF, ECF, FMD, CBPP and AAT were prioritized as the most frequently occurring diseases in cattle herds in the past year. These results affirm previous findings in the region (Kairu-Wanyoike et al. 2014; Kimaro et al. 2017). Important zoonotic diseases such as brucellosis were not identified despite recent reports of high seroprevalence in cattle within the high interface zone (Enström et al. 2017). The fact that this disease was not mentioned by participants could be explained by the lack of awareness or lack of distinct clinical manifestations that allow differential diagnosis with other diseases. The frequent occurrence of prioritized diseases in the target zones is not surprising given the absence of comprehensive vaccination strategies, limited veterinary services, high costs of vaccines and drugs and limited surveillance systems in the area.

The priority diseases were relatively homogenous and strongly agreed between groups of the target zones. The lack of significant differences in the perceived disease risk between zones could be due to limited spatial and temporal resolution considered in this study, which might have been inadequate to show any difference or may be PE tools are not sensitive for determining small changes in risk between contiguous areas (Catley et al. 2012). It is also possible that the unrestricted animal movement between zones (Bedelian and Ogutu 2017) disseminated diseases across the area.

The higher prevalence of FMD in cattle > 4 years than other age groups may be explained by the different grazing strategies used for cattle in various age groups. Weaners and adult cattle > 4 years were more likely to be exposed to FMD virus as they frequently contacted other herds and wildlife during grazing, while for calves, the low prevalence could be due to protective effects of maternally inherited antibodies that wane off as age increases (Elnekave et al. 2016). The median age-specific mortalities for FMD were reported the highest in calves and to decrease with increasing cattle age. The higher mortality in calves could be due to FMD induced myocarditis (Sobhy et al. 2018).

Based on matrix scoring, MCF, FMD, CBPP, anthrax, ECF and AAT had the greatest impact on cattle benefits, mostly on milk production and income from livestock sales. These impacts could have caused huge burden to the households’ dependent on livestock for milk production and income generation on daily basis.

## Conclusion

In general, the dynamic interactions between livestock, wildlife and environment in the livestock-wildlife interfaces promote transmission of multiple infectious diseases. From an ecological perspective, infectious diseases are considered as an ecosystem disservice which pastoralists must consider or trade-off with other benefits such as pasture and water. This study provides information on disease priorities that affect pastoral herds in the defined ecologies, especially where livestock and wildlife interact. Analyses on ecosystem services and trade-offs particularly in pastoral areas should attempt to quantify impacts of multiple diseases that occur in defined localities to obtain more accurate findings. In addition, determining the prevalence of co-infections would also guide the development of more effective interventions including building community’s capacity on disease surveillance for sustained control. Veterinary interventions such as vaccination could also be deployed for multiple diseases. Vaccinations for FMD could, for instance, be conducted together with those of CBPP and ECF provided there are no interferences between vaccines being used. This would drastically reduce the unit cost of deployment of each dose of vaccine. Finally, the differences observed between zones on the prevalence of diseases could be considered while instituting routine disease control programs. Vaccination campaigns could for instance be intensified in zone 1 (with high livestock-wildlife interactions) than in the other areas, e.g. zone 2.

## Electronic supplementary material

ESM 1 (DOCX 16 kb)
