# Supplementary material for: A participatory epidemiological study of major cattle diseases amongst Maasai pastoralists living in wildlife-livestock interfaces in Maasai Mara, Kenya
Source: Trop Anim Health Prod. 2019 Jan 25;51(5):1097–103. doi: 10.1007/s11250-018-01790-1 (PMC6520318; doi:10.1007/s11250-018-01790-1)
Supplement: Supplementary file 1 — (DOCX 16 kb) [file 11250_2018_1790_MOESM1_ESM.docx]

**Supplementary material 1: Overall summarized disease impact matrix scoring results**

| Disease Impact Scoring | FMD  Md (Mn-Mx)^a^ | MCF  Md (Mn-Mx) | Anthrax  Md (Mn-Mx) | ECF  Md (Mn-Mx) | CBPP  Md (Mn-Mx) | Pox  Md (Mn-Mx) | AAT  Md (Mn-Mx) | BEF  Md (Mn-Mx) | Salmonellosis  Md (Mn-Mx) | BCT  Md (Mn-Mx) | Total Md Md(Mn-Mx) |
| --- | --- | --- | --- | --- | --- | --- | --- | --- | --- | --- | --- |
| Reduced milk (*W*=0.16)*** | 4.5 (1-11) | 2.5 (0-6) | 0 (0-4) | 0 (0-3) | 4 (0-20) | 0 (0-2) | 3.5 (0-9) | 0 (0-3) | 0 (0-4) | 0 (0-0) | 1 (0-20) |
| Meat reduction (*W*=0.60)*** | 0 (0-4) | 0 (0,0) | 3.5 (1-11) | 0 (0-2) | 2.5 (0-5) | 0 (1-3) | 0 (0-3) | 0 (0-0) | 0 (0-0) | 0 (0-0) | 0 (0-11) |
| Reduced income  (*W*=0.01)* | 6.5 (0-18) | 10 (0-25) | 4 (0-15) | 2 (0-16) | 7.5 (2-50) | 3.5 (0-9) | 1 (0-8) | 0 (0-5) | 2 (0-2) | 0(0-0) | 3 (0-50) |
| Reduced bride price  (*W*=0.27)*** | 2 (0-9) | 3.5 (0-6) | 2 (0-5) | 0 (0-6) | 2 (0-5) | 1 (0-3) | 0 (0-2) | 0 (0-1) | 0 (0-1) | 0 (0-0) | 0 (0-9) |
| Reduced social status (*W*=0.54)*** | 0 (0-12) | 4 (0-6) | 0 (0-3) | 0 (0-3) | 2 (0-6) | 0 (0-2) | 0 (0-2) | 0 (0-0) | 0 (0-1) | 0 (0-0) | 0 (0-12) |
| Reduced draught power (W=0.89)*** | 0 (0-5) | 0 (0,0) | 0 (0-5) | 0 (0-0) | 0 (0-0) | 0 (0-0) | 3 (0-6) | 0 (0-0) | 0 (0-0) | 0 (0-0) | 0 (0-6) |
| Reduced employment (*W*=0.29)*** | 2 (0-7) | 4 (0-13) | 0 (0-3) | 0 (0-2) | 3 (0-6) | 0 (0-3) | 0 (0-0) | 0 (0-0) | 1 (0-9) | _^b^ | 0 (0-13) |
| Reduced hides for clothing (*W*=0.94)*** | 0 (0) | 0 (0,0) | 2.5 (0-3) | 0 (0-0) | 0 (0-0) | 2 (1-3) | 0 (0-0) | 0 (0-0) | 0 (0-0) | 0 (0-0) | 0 (0-3) |
| Reduced investment | 1 (1,1) | 8 (8,8) | 4 (4,4) | 0 (0-0) | 4 (4,4) | _^b^ | 0 (0-0) | _^b^ | 0 (0-0) | _^b^ | 1 (0-8) |
| Veterinary costs (*W*=0.07)** | 19 (0-30) | 0 (0-16) | 6.5 (0-20) | 11 (0-46) | 33 (9-78) | 0.5 (0-10) | 10 (0-48) | 2 (0-10) | 3 (0-10) | 0 (0-0) | 9 (0-78) |

FMD, foot and mouth disease; MCF, malignant catarrhal fever; ECF, east coast fever; CBPP, contagious bovine pleuropneumonia; AAT, African animal trypanosomiasis; BEF, bovine ephemeral fever; BCT, bovine cerebral theileriosis

^a^ Md, median scores; Mn, minimum; Mx, maximum values are shown in parentheses

^b^ The benefit was not mentioned hence disease impact on the benefit was not scored

*W* = Kendall’s coefficient of concordance in 12 FGDs (*p > 0.05; **p < 0.05; ***p <0.001). Weak agreement, *W* < 0.26, P >0.05; moderate agreement, *W* = 0.26-0.38, P< 0.05; strong agreement, *W* >0.38, P <0.01. *W* value for investment was not calculated due to few cases. The benefit was also not compared by gender and zones.
